# Secondary Metabolites with Antioxidant Activities for the Putative Treatment of Amyotrophic Lateral Sclerosis (ALS): “Experimental Evidences”

**DOI:** 10.1155/2020/5642029

**Published:** 2020-11-24

**Authors:** Jamire M. Silva, Michelangela S. C. Nobre, Sonaly L. Albino, Lucas L. Lócio, Agnis P. S. Nascimento, Luciana Scotti, Marcus T. Scotti, João A. Oshiro-Junior, Maria C. A. Lima, Francisco J. B. Mendonça-Junior, Ricardo O. Moura

**Affiliations:** ^1^Postgraduate Program in Pharmaceutical Sciences-PPGCF, Department of Pharmacy, Federal University of Pernambuco, 50670-901 Recife PB, Brazil; ^2^Drug Development and Synthesis Laboratory, Department of Pharmacy, State University of Paraiba, 58429-500 Campina Grande PB, Brazil; ^3^Postgraduate Program in Pharmaceutical Sciences-PPGCF, Department of Pharmacy, State University of Paraiba, 58429-500 Campina Grande PB, Brazil; ^4^Graduate Program in Chemistry-PPGQ, Department of Chemistry, State University of Paraiba, 58429-500 Campina Grande PB, Brazil; ^5^Laboratory of Cheminformatics, Program of Natural and Synthetic Bioactive Products, Federal University of Paraiba, João Pessoa PB, Brazil; ^6^Laboratory of Synthesis and Drug Delivery, Department of Biological Sciences, State University of Paraiba, 58071-160 João Pessoa PB, Brazil

## Abstract

Amyotrophic lateral sclerosis (ALS) is a fatal motor neuron disorder that is characterized by progressive loss of the upper and lower motor neurons at the spinal or bulbar level. Oxidative stress (OS) associated with mitochondrial dysfunction and the deterioration of the electron transport chain are factors that contribute to neurodegeneration and perform a potential role in the pathogenesis of ALS. Natural antioxidant molecules have been proposed as an alternative form of treatment for the prevention of age-related neurological diseases, in which ALS is included. Researches support that regulations in cellular reduction/oxidation (redox) processes are being increasingly implicated in this disease, and antioxidant drugs are aimed at a promising pathway to treatment. Among the strategies used for obtaining new drugs, we can highlight the isolation of secondary metabolite compounds from natural sources that, along with semisynthetic derivatives, correspond to approximately 40% of the drugs found on the market. Among these compounds, we emphasize oxygenated and nitrogenous compounds, such as flavonoids, coumarins, and alkaloids, in addition to the fatty acids, that already stand out in the literature for their antioxidant properties, consisting in a part of the diets of millions of people worldwide. Therefore, this review is aimed at presenting and summarizing the main articles published within the last years, which represent the therapeutic potential of antioxidant compounds of natural origin for the treatment of ALS.

## 1. Introduction

Amyotrophic lateral sclerosis (ALS), also known as Charcot's or Lou Gehrig's disease, is a progressive and ultimately fatal neurodegenerative disorder characterized by affecting cortical and spinal motor neurons that control the voluntary motions of muscles [1, 2]. It is currently classified according to the mode of the emergence of the pathology in familial (10% of cases), also referred as fALS, inherited in an autosomal dominant, autosomal recessive, or X-linked manner, and sporadic (90% of cases), also known as sALS, which is not genetically inherited [3]. Both types of ALS share the most common symptoms, which manifests as muscle weakness, twitching, and cramping, subsequently leading to the impairment of muscles. In the most advanced stages, the symptoms extend to dyspnea, dysphagia, paralysis, and eventual death from respiratory failure within 3 to 5 years after symptom onset [4, 5].

ALS is the most common motor neuron disease, even though the mechanism evolved in the neurodegenerative dysfunction that leads to motor neurons' loss is not entirely known [6, 7]. Histopathological data shows reduction in neuron size, loss and atrophy of nerve fibers, vacuolization, large empty spaces near neurons, and sponge-like appearance caused by spongiosis [8].

Its incidence is associated with increased age, as it onsets mostly in adults between 50-65 years, and is slightly more prevalent in males than females, with a ratio of approximately 1.6 : 1 [4, 9]. The majority of the epidemiological studies have been conducted in Europe, in which the incidence of ALS is 2-3 people per year per 100,000 general population. Furthermore, an increase in the prevalence of ALS to 8.58 cases per 100,000 inhabitants in the United Kingdom population is expected by the year of 2020 [5, 10].

Multiple pathophysiological mechanisms and cellular dysfunctions are involved as possible causative factors in the disease. Among these, the following stand out: accumulation of reactive oxygen species, mitochondrial dysfunction, neuroinflammation, alterations on RNA processing, glutamate excitotoxicity, apoptosis, protein misfolding and aggregation, autophagy, impaired axonal transport and neurofilament aggregation, and endoplasmic reticulum stress. Moreover, environmental factors have shown to contribute to the pathogeny, such as alcohol, tobacco, physical activity, chemical exposure and metals, and exposure to radiation [1, 10–12].

The oxidative stress is highly correlated with the development and progression of the neurodegeneration in ALS. Changes in the homeostatic balance of ROS production are particularly associated with mutant forms of the antioxidant enzyme SOD1 (encodes for copper/zinc ion-binding superoxide dismutase), which is reported to be the most common causative factor for ALS. Over 150 different mutations have been described in different parts of the enzyme, which include G93A (glycine 93 changed to alanine), H46R (histidine at codon 46 changed to arginine), and A4V (alanine at codon 4 changed to valine). This particular enzyme is responsible for catalyzing the conversion of superoxide (O2^−^) into hydrogen peroxide and oxygen. Its mutation provokes a structural instability that promotes enzyme misfolding, and consequent formation of cytotoxic protein aggregates alone or with other proteins. Consequently, there is a loss of enzyme activity and an increase in the production of ROS that results in deterioration in the regulation of vital cell processes and mitochondrial dysfunction [4, 13–16].

There is no cure for ALS, and current drug treatment is limited since only two drugs are approved by the Food and Drug Administration for the treatment of the disease: Riluzole, a neuroprotective drug that blocks glutamatergic neurotransmission and, more recently, the antioxidant drug Edaravone. These assist in delaying the progression of the disease, extending life expectancy by 2-3 months [2, 17].

Therefore, the obtainment of new drugs, capable of effectively fulfilling the gap in the therapeutic arsenal in order to assist in the treatment and in the decrease of the disease's progression, presents itself as a priority. Among the strategies used for obtaining new drugs, we can highlight the isolation of secondary metabolite compounds from natural products that, along with semisynthetic derivatives, correspond to approximately 40% of the drugs found on the market [18].

Focusing on the influence of ROS for the onset and progression of the disease, the interest in natural compounds with antioxidant potential has become increasing. According to epidemiological studies focused on neurodegenerative diseases, it was observed that there is a significant difference in the incidence of such diseases among ethnic groups with different eating habits, recording that the diet rich in antioxidants is related to a decrease in the incidence rate. Thus, different types of compounds with antioxidant properties can be highlighted, such as the following: polyphenols, which provide protection against ROS and modulate metabolic cascades [19, 20]; carotenoids, which perform antioxidant and neutralizing activities against ROS [19–22]; antioxidant vitamins such as vitamins C and E, among others [13, 14, 23]. Hence, this review is aimed at presenting and summarizing the main articles published within the last years, which represent the therapeutic potential of antioxidant compounds of natural origin for the treatment of ALS. In this sense, the keywords “antioxidant activity,” “amyotrophic lateral sclerosis,” “natural products,” and “secondary metabolites,” among others, were included in the search into the databases “ScienceDirect,” “Pubmed,” “Periódicos Capes,” “Clinical Trials,” “Web of Science,” and “Google Acadêmico,” during the period of March 14 to April 10 2020.

## 2. Secondary Metabolites with Potential Antioxidant Action and Their Possible Applications for ALS

### 2.1. Alkaloids

**Figure 1:**
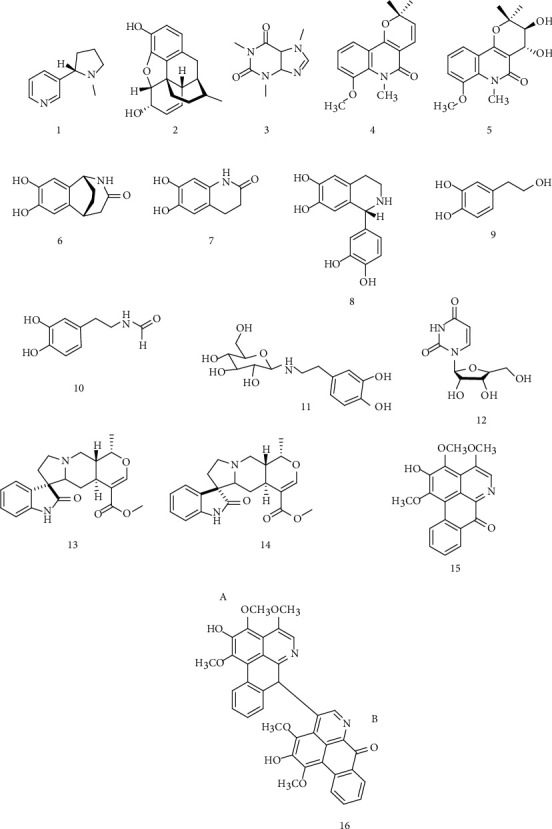
Chemical structure of the well-known alkaloids (nicotine (1), morphin (2), and caffeine (3)), such as those used in preclinical antioxidant studies (8-methoxy-*N*-methylflindersine (4), zanthodioline (5), alternamide A (6), alternamide B (7), alternamine A (8), hydroxytyrosol (9), formamide (10), alternamine B (11), uridine (12), mitraphylline (13), isomitraphylline (14), iraqiine (15), and kareemine (16)).

Alkaloids comprise the largest group of nitrogen compounds, characterized by containing one or more nitrogen atoms within a heterocyclic ring. Over 12,000 compounds are known to be provided by a variety of sources such as animals, bacteria, plants, and fungi [24, 25].  Figure 1 presents the chemical structure of some well-known alkaloids, such as nicotine (1), morphine (2), and caffeine (3). Nitrogen, oxygen, carbon, and hydrogen are the chemical elements that constitute its basic formula, so that its molecular weight varies between 100 and 900 g mol^−1^. This class of compounds has revealed to be one of the most promising secondary metabolites for ALS treatment due to their diverse mechanism of action, which can collaborate to prevent the disease evolution [26–28].

It has been suggested that the increase of extracellular glutamate occurs mainly due to an anomaly in glutamate transporter EAAT2/GLT-1 and leads to ALS. Harmine, a beta-carboline alkaloid, may act by activating EAAT2/GLT-1 gene expression increasing the cellular uptake of glutamate [29]. The indirect mechanism was suggested by the use of alkaloids with commercial drug Riluzole, aiming to block the increased levels of P-glycoprotein in the blood-brain barrier (BBB) [30]. In this sense, Lopez and Martinez-Luis showed that polyaromatic alkaloids from *prosobranch* mollusk (*Lamellaria* sp.) restrain P-gp, demonstrating an increased cellular uptake to different drugs in resistant cell line [31]. However, in this review, we will highlight the benefits of its mechanism of action as antioxidants, since alkaloids have the ability to convert free radical species in inactive substances [32, 33]. Thus, researches have explored the advantages of employing alkaloids with this purpose, alone or included in a pharmaceutical form and from several sources [34–37].

For example, in order to evaluate the antioxidant activity of two quinoline isolated alkaloids from *Zanthoxylum rhetsa* root bark, Zohora and collaborators showed through DPPH assay that the compounds 8-methoxy-*N*-methylflindersine (4) and zanthodioline (5) (Figure 1) presented free radical scavenging activity (IC_50_ in *μ*g/mL) of 71.18 ± 1.74 and 101.90 ± 5.24, respectively, reveling potent antioxidant activity [38].

*Alternanthera littoralis P. Beauv*. is a tree common in tropical, subtropical, and temperate regions. Koolen et al. isolated seven chemical alkaloids from the aerial parts of this plant, which include two known alkaloids, hydroxytyrosol (9) and uridine (12), and five new alkaloids, namely, alternamide A (6), alternamide B (7), alternamine A (8), N-(3,4-dihydroxyphenethyl) formamide (10), and alternamine B (11) (Figure 1). The biological evaluations showed the high antioxidant free radical scavenging effect of the ethanolic extract containing alkaloids alternamide B (1.10 relative Trolox equivalent—RTE), alternamide A (0.85 RTE), and alternamine B (0.65 RTE) in relation to positive control quercetin (5.62 RTE) [39].

Azevedo et al. studied the alkaloids mitraphylline (13) and isomitraphylline (14) (Figure 1) obtained from extracts of the aqueous leaf extract (ALE) from *Uncaria tomentosa* which present high phenolic content (153.51 *μ*g gallic acid equivalents per milligram extract). The ALE *in vitro* antioxidant activity investigation, performed using DPPH, ABTS, and ferric reducing antioxidant power (FRAP) assays, obtained values of IC_50_ = 10.68 ± 0.64 *μ*g mL^−1^, 2.15 ± 0.29 mmolTrolox/mg AA and extract, and 2.9 ± 0.15 mMFeSO_4_/mg AA and extract, respectively, presenting a moderate activity [40].

Aiming to obtain new alkaloids, Aldulaimi and collaborators isolated from *Alphonsea cylindrical King* two novel isoquinoline alkaloids, namely, iraqiine (15) and kareemine (16) (Figure 1). *In vitro* antioxidant activity was performed, and the results showed IC_50_ values of 48.77 and 101.66 *μ*g mL^−1^, respectively. In this same test, five additional known alkaloids were tested and among them, O-methylmoschatoline (144.15 *μ*g/mL) was better than kareemine [41].

Nevertheless, despite of examples above, researching for papers about this matter, using “antioxidant activity” and “alkaloids” as keywords on the Scholar, with range of last 5 years, it is possible to found 822 papers that cite these terms in the abstract and/or title [42]. When the term “ALS” is added, no articles are found and similar results are observed in other databases (Web of Science, Pubmed, and ScienceDirect). These works always present alkaloids obtained from different sources and evaluate their biocompatibility and antioxidant activity *in vitro* using different methods [43, 44]. The association with ALS always happens in a generalized way because one treatment option to neurodegenerative diseases is using this approach [45].

The lack of studies in the literature performing specific preclinical or clinical tests for ALS and no therapy consolidate to alkaloids despite advantages can be by two main problems, not to easy standardization in batch-to-batch reproducibility and scale-up [46]. For these reasons, the use of isolated alkaloid molecules can circumvent these drawbacks. Another possibility is alkaloids-loaded in nanoscale systems to develop new formulation in terms of tissue/organ targeting and BBB penetration as shown in literature [47, 48].

Finally, alkaloids can slow down ALS by three hypothetical mechanisms of action, as mentioned above. Several studies evidenced satisfactory results in terms of antioxidant activity; thus, clinical trials (phase I) are necessary in order to better understand the previously mentioned mechanisms and advance this treatment proposal to improve patients' life quality.

### 2.2. Flavonoids

**Figure 2:**
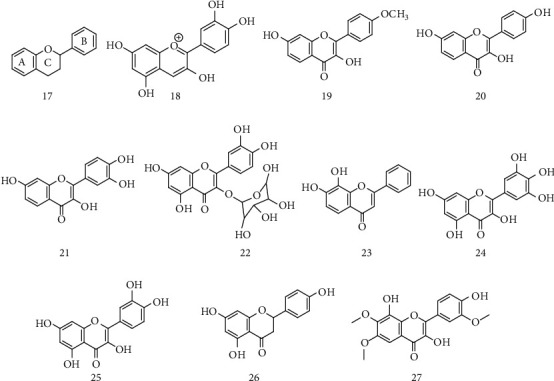
Structure of flavin (17) and anthocyanin (18), kaempferida (19), kaempferol (20), fisetin (21), compound 22, 7,8 dihydroxy flavone (23), myricetin (24), quercetin (25), naringin (26), and 3,5,4′-trihydroxy-6,7,3′-trimethoxy flavone (27).

Flavonoids are phenolic compounds derived from the aromatic amino acids phenylalanine and tyrosine, which are widely found in plants. Its structure consists of a flavin nucleus (17) containing 15 carbon atoms arranged in three rings (C6-C3-C6), labeled as A, B, and C, as illustrated in Figure 2. These compounds have a broad pharmacological spectrum, in which their antioxidant and anti-inflammatory stand out, which mainly associated with the fight against neurodegenerative diseases such as ALS [49].

It is known that processes such as oxidative stress, neuroinflammation, and neural apoptosis collaborate with ALS progression; in this sense, Winter et. al. evaluated the effects of strawberry extract enriched with anthocyanin (18) (Figure 2) in mice with ALS model (G93ASOD1 mutation), as this class of flavonoids has antioxidant, anti-inflammatory, and antiapoptotic activity established in previous studies [50–52]. The trials showed that at a dose of 2 mg/kg/day, guinea pigs suffered a marked delay of approximately 17 days at the onset of the disease, in addition to an increase in life expectancy of approximately 11 days. Histological analysis exhibited a significant reduction in spinal cord injury when compared to mutant guinea pigs not treated with the extract. For that reason, this study emphasizes that anthocyanins have therapeutic potential for this disease and can evolve to more complex pharmacological tests [53].

There are reports in the literature suggesting that the mutation in superoxide dismutase (SOD1) at position 85 of glycine and arginine is a fundamental cause for the onset of ALS. Taking advantage of the antioxidant activity of the flavonoids kaempferida (19) and kaempferol (20) (Figure 2), the study performed by Srinivasan and Rajasekaranb evaluated through molecular docking their aggregation affinity towards SOD1. Kaempferol portrayed stronger hydrophobic interactions with the target in comparison to kaempferida, thus qualifying it as a possible prototype for the design of new drugs for the treatment of ALS [54].

Ueda and collaborators also proved the action of kaempferida and kaempferol, extracted from Brazilian green propolis, as a possible alternative for the treatment and prevention of ALS. In this study, kaempferida (15 *μ*M) and kaempferol (3.0 *μ*M) were tested against Na^2+^ cells (superoxide induced) with mutant SOD1. The results showed significant inhibition of mutant SOD1, as well as in Western blot analysis, which showed that kaempferol induced autophagy through the protein kinase activated by AMP, suggesting this as a probable mechanism for neuroprotection and emphasizing this compound as a good therapeutic alternative on the treatment of ALS [55].

A flavonoid known as fisetin (21) (Figure 2), a natural antioxidant, already presented several benefits in some degenerative diseases such as Alzheimer's and Parkinson's. Therefore, Wang et al. performed *in vitro* assays on mutant cells (hSOD1G85R and hSOD1G93A) and *in vivo* assays with hSOD1 transgenic mice in order to evaluate the efficiency of the compound to SOD1 associated with ALS. Treatment with fisetin improved motor impairment, prolonged life span, and confirmed the neuroprotective antioxidant effects, proposing this flavonoid as a strong drug candidate with therapeutic value for the treatment of ALS [56].

Studies in mice revealed that the first steps in the development of ALS may comprehend the increased presence of SOD1 monomers and their aggregation to motor neurons. In this perspective, Ip and collaborators carried out tests *in silico* with a library of 4,400 drugs and natural compounds that were coupled in the SOD1 dimer. Between these compounds, seven with best results were tested for aggregation and splitting of SOD1 induced by hydrogen peroxide in vitro at concentrations ranging from 20 *μ*M to 100 *μ*M. Compound 22 (Figure 2) revealed that quercetin-3-*β*-D-glucoside presented one of the best results, and for that, it may be a potential therapeutic inhibitor of misfolding and aggregation of SOD1 and, therefore, can delay clinical symptoms of ALS [57].

The use of *Panax ginseng* for the alternative treatment of ALS is recurrent, but without scientific proof, these effects may be related to the increase in the expression of the growth factor for cells of the nervous system and high antioxidant activity. For these reasons, Jiang et al. studied the effects of ginseng extract, rich in flavonoids, at doses of 40 and 80 mg/kg in B6SJL-TgN (SOD1G93A) transgenic mice. Results showed a delay in the appearance of diseases' signs and increase in survival [58], pointing out the potential of this root and possible mechanism action.

Korkmaza et al. observed that the chronic administration of 5 mg/kg of flavonoid 7,8-dihydroxyflavone (23) (Figure 2), generally found in the plants *Godmania aesculifolia* and *Tridax procumbens*, in mice with ALS (SOD1G93A) by up to 105 days caused a significant improvement in motor deficits. This result is believed to be associated with the compound's ability to cross the blood-brain barrier and exercise neuroprotective activity therefore can decrease the clinical manifestations of the disease [59].

As previously mentioned, the formation of poorly folded protein aggregates inside or outside neural cells is one of the causes of neurodegenerative diseases, such as ALS. In this sense, Joshi and collaborators presented, through *in vitro* studies with Cos-7 cells in immunofluorescence staining assay with primary antibodies of E6-AP and Hsp70, that at a concentration of 15 *μ*g/mL, mycetin flavonoid (24) (Figure 2) is capable to eliminate or suppress the aggregation of different proteins and reduce the inclusion of folded proteins and leading to a decrease in the symptoms of muscle paralysis [60].

Excessive neuronal stimulation of glutamate leads to a large influx of Ca^2+^ which, when accumulated in mitochondria, triggers an increase in the production of reactive oxygen species for the formation of oxygen free radicals, just as this excitation leads to the activation of caspase3, which seem to be involved in the etiology of many neurodegenerative disorders, such as ALS. In this context, Shimmyo et al., through *in vivo* studies with mice induced by glutamate and *in vitro* tests with neuronal cells, showed that myricetin 24 (Figure 2) inhibited the glutamate-induced excitotoxicity by three routes: reduction of intracellular calcium through the phosphorylation of NMDAR (*N*-methyl D-aspartate receptor), inhibition of free oxygen radicals, and inhibition of capase-3 [61].

Aluminum has been associated with several neurological diseases, including Alzheimer's disease, Parkinson's disease, and ALS; it is suggested that this neurotoxicity may be associated with the fact that aluminum is capable of generating reactive oxygen species (ROS) [60]. In this perspective, Sharma et al. [62] evaluated the antioxidant activity of quercetin (25) (Figure 2), at a dose of 10 mg/kg body weight/day in mice whose oxidative stress was induced by aluminum (10 mg/kg of body weight/day). The results revealed that quercetin decreased neuronal apoptosis and decreased the production of free radicals, in addition to obstructing neurodegenerative histological changes induced by aluminum.

Zhuang et al. analyzed through mass spectrometry and molecular docking the noncovalent interactions between SOD1 and selected flavonoid compounds, such as quercetin, rutin, naringin, and hesperidin, among others. The study is aimed at identifying which compound has a more prominent capacity to inhibit the aggregation of the apo-SOD1 complex. Through these techniques, the results showed that naringin (26) (Figure 2) presented better aggregation inhibition profile, being considered as an interesting compound for the treatment of ALS [63].

As mentioned in the literature, glutamate toxicity is a major contributor to the appearance of neurodegenerative diseases such as ALS and Alzheimer's disease. Hence, Elmann and collaborators [64] isolated the sesquiterpene lactone achillolide A and the flavonoid 3,5,4′-trihydroxy-6,7,3′-trimethoxyflavone (27) (Figure 2) from the *Achillea fragrantissima* plant and tested in Na^2+^ cells of neuroblastoma of mice intoxicated with glutamate. The results show that the compounds protected astrocytes from cell death induced by oxidative stress and inhibited microglia activation, demonstrating its significant protective effect. In this sense, we highlight the importance of flavonoids as a therapeutic potential tool against the decrease in the time of onset of symptoms and the increased survival of patients with ALS, whether by antioxidant, neuroanti-inflammatory, and antiprotein aggregation mechanisms.

*In silico* and *in vivo* tests of the cited studies demonstrated that extracts enriched with flavonoids, as well as their isolated, may act in the treatment of ALS by different mechanisms of action, such as decreased spinal cord injury, SOD1 inhibition, inhibition of motor neuron aggregation, inhibition caspase3, and decrease reactive oxygen species. These compounds may cause a delay in the appearance of the disease's signs and decrease the clinical manifestations. Therefore, by evaluating the toxicity of these compounds and establishing a therapeutic dose, they could potentially be qualified to undergo more advanced pharmacological tests, such as clinical trials.

### 2.3. Coumarins

**Figure 3:**
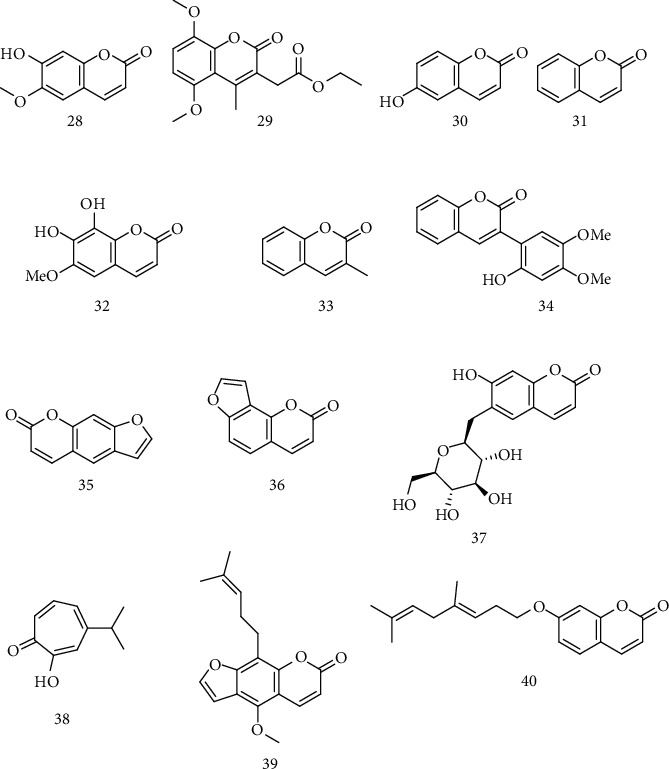
Structure of coumarins scopoletin (28), trigocoumarin (29), esculentin (30), coumarin (31), fraxetin (32), methyl-3-coumarin (33), caraganolide A (34), psoralen (35), isopsoralen (36), esculin (37), hinokitol (38), felopterin (39), and auraptene (40).

Coumarins are another class of secondary metabolites implemented in the literature as promising molecules due to their results against neurodegenerative diseases such as ALS. Based on these facts, Mogana et al. tested the antioxidant capacity *in vitro* of scopoletin (28) isolated from *Canarium patentinervium* (Figure 3), starting from chloroform extract and purification by silica gel chromatography using the hydrogen atom transfer (HAT) and simple electronic transfer (SET) method. This coumarin presented strong antioxidant potential (EC_50_ 191.51 ± 0.01 *μ*M), demonstrating its ability to act in diseases caused by oxidative stress [65].

*In vitro* studies for DPPH, hydroxyl, and ABTS radical scavenging activity carried out by Kumar et al. to determine the antioxidant capacity of substances present in fenugreek seed extract (*Trigonella foenum-graecum*), such as trigocoumarin (29) (Figure 3), were performed. The antioxidant activity of the extract was expressed in IC_50_ and showed its efficiency in reducing DPPH free radicals, being able to reduce them by 50% at a concentration of 395 *μ*g/mL (*R*^2^ = 0.9712) [66].

Oxidative stress was induced in NSC34 motor neurons of rats by Barber and collaborators in the presence and absence of several compounds. Among them, the coumarin esculentin (30) (Figure 3) was the only one of the studied compounds capable of directly buffering the radicals, promoting protective effects for cells. This article does not present the form of obtainment of these compounds [67].

Hasnat and collaborators presented results for inhibition of acetylcholinesterase, as well as *in vitro* and *in vivo* antioxidant activity of *Ganoderma lucidum* that grows in germinated brown rice (GLBR), in which coumarin (31) (Figure 3) is presented as the fourth most predominant compound between the thirteen identified by HPLC analysis of the GLBR extract. *In vitro* results revealed the occurrence of inhibition of peroxidation induced in brain cells of rats by Fe^2+^. However, in *in vivo* results, no significant variations could be observed when doses of 100 mg/kg were administered in the treatment group [68].

Li and Seeram identified the presence of seven lignans, as well as two coumarins isolated from the butanol extract of the maple syrup (MS-BuOH), being identified as scopoletin and fraxetin (32) (Figure 3). These coumarins were evaluated for their antioxidant activity and obtained IC_50_ values of 68.2 ± 31.2 and 46.5 ± 3.6 *μ*g/mL, respectively. Therefore, these compounds better result in comparison to the seven isolated lignans (values ranging between 101.5 ± 5.9 and 1335.9 ± 47.6 *μ*g/mL) and stilbenes contained in sugar fraction of maple syrup (>2600 *μ*g/mL). Moreover, fraxetin also showed a better result when compared to vitamin C (IC_50_ = 58.6 ± 10.7 *μ*g/mL) [69].

Glutamatergic excessive transmission is associated with several neurodegenerative diseases such as ALS. In order to analyze the antioxidant and neurodegenerative capacity of the seed extract of *Amburana cearenses*, Pereira and collaborators promoted studies in PC12 cell cultures which were induced by glutamate excitotoxicity. Among the isolated compounds, methyl-3-coumarin (33) was identified (Figure 3). In the isolate containing mainly coumarins, including compound 33, promoted, after 24 h, a reduction of 17% in death induced by glutamate, in addition to inducing neuroprotection in 1000 *μ*g/mL [70].

To test the ability to inhibit oxidative stress from the extract of *Angelicae dahuricae radix*, which presents furanocoumarin as a secondary metabolite, Moon and colleagues examined its effect on reducing the level of ROS in BV2 cells activated by lipopolysaccharides (LPS). It was observed a significant decrease in ROS levels at the concentrations of 10 and 50 *μ*g/mL. *In vivo* tests were performed using a dose of 100 mg/kg administered orally to rats during 14 days. The results indicated the extract's neuroprotective effect after spinal cord injury. In this way, the extract has a possible neuroprotective capacity for diseases such as ALS, mainly due to the decrease in cytokine levels when damage to BV2 cells is inferred [71].

A plant of the same genus, *Angelicae gigas*, was evaluated for its antioxidative effect directed to neurodegenerative diseases by Park and collaborators, where the presence of coumarins in this plant has been identified in previous works published by Ryu et al. [72]. *In vitro* tests on DPPH and determination of the reducing capacity demonstrated that the ethanol extract showed an EE_50_ of 31.47 ± 0.68 mg/mL in the elimination of DPPH. The ethanol extract also showed strong antioxidant activity with an IC_50_ of 43.22 ± 1.67 *μ*g/mL, demonstrating to be more effective in comparison to the aqueous and methanolic extracts, and to the positive control *α*-tocopherol (68.64 ± 5.47 *μ*g/mL). The initiation and propagation of free radicals in the body can be related to transition metals in which elements such as iron and copper act as powerful catalysts in oxidation reactions. Complementing the tests, metal chelating activity was carried out, which consists of adding different concentrations of the extract, methanol, and FeCl_2_. For the ethanolic extract, an accentuated binding capacity with Fe^2+^ was verified [73].

The effects of *Caragana turfanensis* on neuroinflammation inhibition were evaluated by Song et al. Among the 36 compounds present in the extract, a new coumarin, caraganolide A (34), was observed (Figure 3). The evaluation of each compound was performed on LPS-induced BV2 cells, and caraganolide A demonstrated an IC_50_ of 1.01 ± 1.57 *μ*g/mL, portraying the best activity among all isolated compounds, including in comparison to the positive control (minocycline) of 9.07 ± 0.86 *μ*g/mL [74].

From the extract of aerial parts from *Ducrosia ismaelis asch*, Morgan and collaborators were able to isolate psoralen (35) and isopsoralen (36), among other compounds (Figure 3). *In vitro* antioxidant activity of these coumarins demonstrated that the elimination of peroxy radicals and the reducing capacity were inferior to the other components of this extract; however, the inhibition of soluble epoxide hydrolase (sEH) by psoralen was favorable, indicating that this compound can act improving the antioxidant defense mechanism, thus being considered a promising compound for diseases such as ALS [75].

Varier et al. performed molecular docking, using Auto Dock tools 1.5.6 program and the MGL tools 1.5.6 package, to predict the inhibitory capacity of esculin and hinokitol. In the target monooxidase b, esculin (37) (Figure 3) obtained a more favorable binding affinity, with free energy result of -9.6 Kcal/mol, while the cocrystallized ligand levodopa and hinokitol (38) presented binding energies of -7.7 Kcal/mol and -7.2 Kcal/mol, respectively. For all six evaluated different targets, esculin exhibited the best results in comparison to the aforementioned ligands. Monooxidase B is found in the external mitochondrial membrane, whose dysfunction of this organelle may be associated with common neurodegenerative diseases such as ALS and Alzheimer's disease [76].

In neurodegenerative diseases such as ALS, compounds that activate signal-regulated kinase phosphorylation (ERK) and element response-binding protein (CREB) are considered as possible neuroprotective agents. Based on this information, Nakamura et al. tested phosphorylation capacity in human A172 cells of felopterin (39) and auraptene (40) isolated from citrus fruits (Figure 3). Both coumarins promoted an activation of ERK and CREB, indicating that the coumarin ring is a possible pharmacophore responsible for activating the neuroprotective effect on the central nervous system [77].

In general, there is an improvement in coping with neurodegenerative diseases such as ALS through the use of isolated coumarins or plant extracts containing them. The results were promising when performed *in silico*, *in vitro*, and experimentally *in vivo*, revealing its antioxidant effect, prolongation of the programmed death of neural cells, and neuroprotection, beyond the reduction of free radicals.

Most coumarins from natural products could potentially be tested for antioxidant activity, reduction of DPPH, inhibition of induced peroxidation, reduction of glutathione-induced death, and induction of neuroprotection. In general, there is an improvement in dealing with neurodegenerative diseases, such as ALS, through the use of isolated coumarins or plant extracts containing them. The results were promising when performed in silico, in vitro, and experimentally in vivo, revealing its antioxidant effect, prolongation of the programmed death of neural cells, and neuroprotection, beyond the reduction of free radicals.

### 2.4. Other Metabolites

In addition to the secondary metabolites present in natural products with antioxidant action already mentioned for the treatment of ALS, it is also common the presence of other metabolites in extracts or fractions of extracts present in leaves, fruits, and roots, as well as in other natural products such as fungi and algae [78–80]. From here, we will present the presence and antioxidant mechanisms of tannins, terpenoids, lignans, quinones, saponins, methylxanthines, glucosinolates, and fatty acids directed to the treatment of ALS.

### 2.5. Tannins

Tannins are secondary polyphenolic plant metabolites commonly found in extracts from a wide variety of plants [81, 82]. Studies of extracts of several plants containing tannins have already reported their biological activity for the treatment of various diseases, such as neurodegenerative diseases, diabetes, lung infections, rheumatism, and kidney diseases, among others [83, 84].

In this perspective, Auddy and collaborators evaluated through *in vitro* and *in vivo* tests, in an unprecedented way, the toxicity and the effect of eliminating radicals with ethanolic and aqueous extracts of three plants commonly used in Indian medicine: *Sida cordifolia*, *Evolvulus alsinoides*, and *Cynodon dactylon*. The results demonstrated that the water infusion of the three plants (up to 1 mg/mL) did not show toxic effects on the PC12 cell line in the MTT tests. As for the verification of antioxidant effects, it was shown that in the ABTS test, the ethanolic extract of *S. cordifolia* presented a better result, with an IC_50_ of 16.07 *μ*g/mL, followed by *E. alsinoides* and *C. dactylon*, with IC_50_ values of 33.39 *μ*g/mL and 78.62 *μ*g/mL, respectively. As for water infusions, the IC_50_ values were, respectively: 172.25 *μ*g/mL for *E. alsinoides*, 273.64 *μ*g/mL for *C. dactylon*, and 342.82 *μ*g/mL for *S. cordifolia*. In the results of lipid peroxidation (TBARS), the water infusion of *E. alsinoides* demonstrated the best result out of the three infusions with an IC_50_ of 89.23 *μ*g/mL. *In vivo* studies have not shown significant results, and the author attributes the experimental model as being ineffective due to the high, rapid, and extensive degradation of the antioxidant inside the body. The results were promising for the continuity of tests and verification of activity in neurodegenerative diseases [85].

Banerjee et al. reported an *in vitro* study that evaluated the antioxidant activity of black plum peel and juice extract scientifically known as *Syzygium cumimi* of the *myrtaceae* family. The antioxidant activities of the aqueous extracts were verified for hydroxyl radicals, superoxide radicals, and stable free radicals 1,1-diphenyl-2-picryl-hydrazil (DPPH). The IC_50_ values for the fruit peel were 468 *μ*g/mL, 260 *μ*g/mL, and 168 *μ*g/mL, respectively. Thus, it can be said that the antioxidant activity of *S. cumini* has significant antioxidant activity. The author attributes this activity to the presence of tannins in the extracts and the peel of the fruit may be important for further studies in the fight against diseases, including ALS [86].

The antioxidant activity of polyphenols is mainly linked to its redox properties and hydrogen donation [87, 88]. Aware of these properties, Mahesh and collaborators carried out studies to evaluate the antioxidant effect and of a plant native to India, *Terminalia chebula* (*combretaceae*), traditionally used to treat various diseases in Asia. The elimination of superoxide radicals, hydroxyl radicals, and nitric oxide radicals with the aqueous extract of the fruit peel of *T. chebula* was evaluated showing significant results with IC_50_ values of 0.031 mg/mL, 0.097 mg/mL and 0.744 mg/mL, respectively. Thus, although the tannin fraction has not been isolated, the author attributes a greater contribution to the antioxidant properties. It also highlights the importance of studying *T. chebula* for possible applications in the treatment of neurodegenerative diseases [89].

A plant-based multicomponent, called Padma® 28, contains, in addition to other compounds, flavonoids and tannins. It has had its antioxidant activity reported by Ginsburg and collaborators, who also evaluated the neuroprotective activity of the extract in a PC12 neuronal cell strain against the toxins A*β*25-35 (10 mM), glutamate (40 mM), MPTB (5 mM), and 3-NP (10 mM). Faced with these neurotoxins, PC12 cells demonstrated reduced mitochondrial dysfunction following Padma 28 treatment, thus decreasing cellular oxidative capacity. The decrease induced by Padma® 28 can be attributed to the direct elimination of reactive oxygen species generated in PC12 cells or by the interference with the generation of hydroxyl radicals [90].

Chang and colleagues evaluated *in vitro* the antioxidant activity and neuroprotective effects of five methanolic extracts from dry parts of the plants *Spatholobus suberectus* (SSE), *Uncaria rhynchophylla* (URE), *Alpinia officinarum* (AOE), *Drynaria fortune* (DFE), and *Crataegus pinnatifida* (CPE). Analyzing the extracted fractions, it was verified that the SSE extract contained a higher content of flavonoids, while the URE and AOE extracts contained a higher percentage of tannins and triterpenoids, respectively. Their antioxidant effects were evaluated by 4 means: HRP-luninol-H_2_O_2_, pyrogallol–luminol, CuSO4-Phen-Vc-H_2_O_2_, and Luminou-H_2_O_2_. The URE extract demonstrated good antioxidant activities in the HRP-luninol-H_2_O_2_ and CuSO_4_-Phen-Vc-H_2_O_2_ assays, with respective IC_50_ values of 3.6 ± 0.4 *μ*g/mL and 5.9 ± 0.5 *μ*g/mL. These values approximated and, in some cases, better than, the positive controls: vitamin C (14.8 ± 6.2 *μ*g/mL and 688.3 ± 29.7 *μ*g/mL) and Trolox (3.2 ± 0.1 *μ*g/mL and 5.8 ± 1.0 *μ*g/mL) [91].

Regarding the neuroprotective evaluation, the neurogrowth effects in H_2_O_2_ of the five extracts in PC12 were evaluated. The extracts that had the best neuroprotective effects were CPE, URE, and SSE in the concentrations ranging from 0.5 to 5.0 *μ*g/mL, with a percentage of neuroprotection from 78.1 to 107.2% for CPE, 54.1 to 85.7% for URE, and 46.3 to 65.7% for SSE, compared to the positive control AC-DEVDCHO (N-acetyl-Asp-Glu-Val-Asp-al) with values from 23.9 to 37.5%. Therefore, the extracts have the potential to be deepened into *in vivo* studies for the treatment of neurodegenerative diseases, in which ALS is included [91].

In the study conducted by Adewale and colleagues with the leaves of *Solanum macrocarpon*, a plant typical of India, had its antioxidant activity verified in the protection of the tissues of rats, especially the liver and brain, against iron-induced lipids (Fe^2+^). Aqueous extracts showed levels of flavonoids and phenolic compounds (with the presence of tannins). The results demonstrated that the aqueous extract of the leaves of *S. macrocarpon* showed high inhibition of lipid peroxidation induced by iron sulfate II with an inhibition value of 92.91 ± 1.56% at a concentration of 3.33 *μ*g/mL, showing to be more effective than the quercetin control (concentration of 90.15 ± 1.35 *μ*g/mL). Thus, the aqueous extract of the leaves of *S. macrocarpon* presented with a strong antioxidant agent, offering protection against oxidative damage of liver and brain tissues, which places it as a potential for future analyses in the treatment of ALS and neurodegenerative diseases [92].

Another *in vitro* study was carried out by Hamamcioglu and collaborators, in which the evaluation of the potential of the neuroprotective effect in PC12 (dPC12) cells, as well as the antimutagenic and antigenotoxic effects of the species of the plant *Glaucium acutidentatum* of the *papaveraceae* family was carried out. The methanolic extract, at a concentration of 500 *μ*g/mL, showed better results for cell damage, caused by H_2_O_2_, with values between 80 and 90% of neuroprotection, in comparison to the aqueous extract that presented a rate between 65 and 75%. It was also observed that the aqueous extract presented no cytotoxicity (90 to 96%), while the methanolic extract presented moderate cytotoxicity (67 to 75%) at any concentration. Another important data demonstrated in the same study is concerning the neuritis index, which is strongly linked to the neuroprotective effect. The higher the value, the better the neuroprotective result [93], as it indicates neural regeneration. It was found that in the presence of only H_2_O_2_, neuritis concentration dropped to 50.7 ± 0.4 *μ*M, while the control untreated cells presented a value of 80.3 ± 0.3 *μ*M and the extracts (methanolic and aqueous), in a concentration of 500 *μ*g/mL, exhibited values of 78.8 ± 0.5 *μ*M and 78.2 ± 0.3 *μ*M, respectively. Furthermore, the extracts showed antimutagenic activity (75.0 and 74.8% inhibition) and antigenotoxic activity. This is a pioneering study for antigenotoxic activity. The results demonstrate strong candidates for the treatment of multiple degenerative diseases, including ALS [94].

The studies presented above with extracts containing tannins in the treatment of ALS demonstrate the lack of in-depth studies, considering that they do not perform in vivo experiments, therefore limited to in vitro studies, and, consequently, there are no subsequent preclinical tests and specific clinical trials for ALS. Possibly this continuity of the experiments is linked to the isolation and quantification of the compounds, considering that the majority of the studies did not present gas chromatography techniques.

### 2.6. Terpenoids

**Figure 4:**
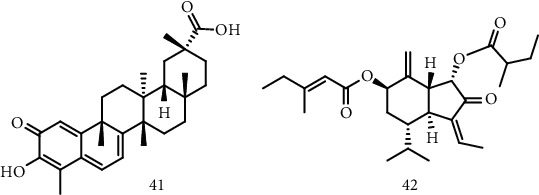
Structure of celastrol (41), a natural triterpenoid isolated from a Chinese plant *Tripterygium wilfordii*, and ECN (42), isolated from the plant *Tussilago farfara.*

Terpenoids are terpenic compounds that have undergone oxidation. Many studies have been reported with the use of terpenoids in the treatment of diseases [95–97]. One of these studies was carried out by Kiaei and collaborators through the evaluation of the neurodegenerative activities of celastrol (41) (Figure 4), a triterpenoid, isolated from a Chinese plant *Tripterygium wilfordii* of the family *Celastraceae.* In this study, the neuroprotective effects were examined in the G93A SOD1 transgenic mice model for ALS. Studies have shown that the treatment with celastrol significantly improved motor function, delaying ALS symptoms, and also causing a decrease in mice's weight. Another relevant result was the increase in survival of the G93A mice treated with celastrol, registering 9.4% and 13% at the dosages of 2 mg/kg/day and 8 mg/kg/day, respectively. Moreover, an increase of 30% of the neuronal concentration in the lumbar spine suggests a protective effect. Histological analysis revealed a reduction in TNF-*α*, iNOS, CD40, and GFAP immunoreactivity. Thus, celastrol can be a promising therapeutic agent for the treatment of ALS [98].

*In silico* and *in vivo* evaluations of phytochemicals present in the root extract of the *Asparagus adscendens* (AAE) plant of the *Liliaceaee* family were carried out by Pahwa and Goel, aiming to evaluate the nootropic, antiamnesic, and antioxidative activities. Prediction of Activity Spectra for Substances (PASS) and Pharmaexpert were used for *in silico* studies. *In vivo* studies of antiamnesic activity were assessed by scopolamine induction. Finally, nootropic activity was assessed by evaluating the effect of the extract of AAE against antioxidant enzymes on the cortex and hippocampus of mice and acetylcholinesterase. The extracts showed, in addition to other secondary metabolites, the presence of terpenoids. The results *in silico* pointed to a significant decrease in memory loss that was proven by *in vivo* studies. Besides, the AAE extract considerably reduced the levels of oxidative stress in the cortex and hippocampus of mice [99].

Lee et al. investigated the neuroprotective effects of a sesquiterpenoid, ECN (7*β*-(3-ethylcis-crotonoyloxy)-1*α*-(2-methylbutyryloxy)-3,14-dehydro-Z-notonipetranone) 42) (Figure 4) obtained from *Tussilago farfara* sprout, from the *Asteraceae* family, mediated by Nrf2 (nuclear factor erythroid 2) against oxidative stress in the PC12 cell line, as well as the potential of ECN to activate Nrf2 inducing OH^−1^, also observing the protective effects of ECN (10 *μ*M) in an experimental model of animals with neurodegeneration. The results demonstrated a protective effect against H_2_O_2_ and 6-hydroxydopamine (6-OHDA) with 91.8 ± 6.6% and 87.9 ± 1.7% inhibition in the concentration of 500 *μ*M of H_2_O_2_ and 250 *μ*M of 6-OHDA, respectively. The ECN also showed good results in the positive regulation of ARE-luciferase activity (cell line designed to monitor the induction of antioxidant response elements), as well as in the induction of Nrf2 and OH^−1^ mRNA expression. The results also suggested an interaction between ECN and Keap1 modifying the cysteine thiols of Nrf2, causing a positive regulation of OH^−1^. Furthermore, the administration of ECN improved dopaminergic and motor neuronal damage [100].

### 2.7. Lignans

**Figure 5:**
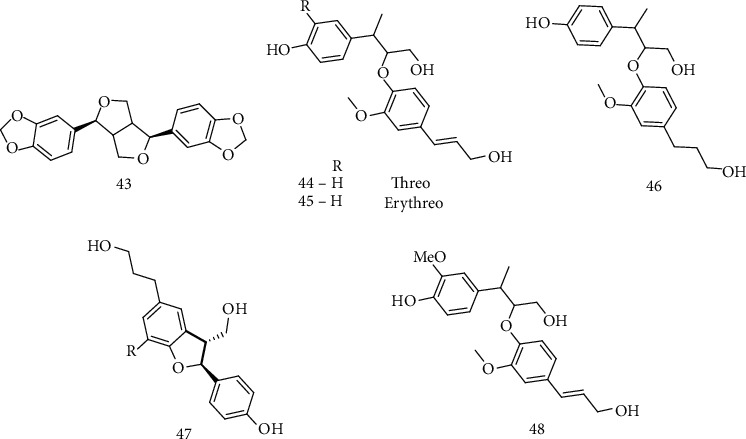
Structures of sesamine (43), a lignan isolated from sesame seed (*Sesamum indicum L*) and other lignans (44, 45, 46, 47, and 48) isolated 12 lignans from the fruit of the plant *Broussonetia papyrifera.*

Lignans are a group of phytochemicals, which are produced by oxidative dimerization of two phenylpropanoid units [101]. Studies related to the use of plant extracts containing lignans have also been reported in the literature for the treatment of neurodegenerative diseases [102, 103] mainly by the mechanism of peroxidation dismutase-SOD1. In one of these reports, Lahaie-Collins et al. evaluated sesamine (43) (Figure 5), a lignan isolated from sesame seed (*Sesamum indicum L*) and bark of *fagara* plants, for neuroprotective, antioxidant, and anti-inflammatory effects on neuronal cells PC12. The compound 1-methyl-phenylpyridine (MPP+) was used as an oxidative damage inducer. The results showed that in picomolar doses (10^−12^ M), sesamine protected the PC12 neuronal cells from oxidative stress, attenuating by up to 60% the cell death caused by MPP+, in addition to reducing the production of reactive oxygen species. Further results revealed an increase in the production of SOD proteins, as well as a reduction in catalase. Sesamine showed potential in preventing neurodegenerative diseases [104].

Mei et al. isolated 12 lignans from the fruit of the *Broussonetia papyrifera* plant, among which 9 were unpublished. The ethanolic extracts of each lignan were evaluated for its antioxidant activities via MTT and DPPH in PC12 neuronal cells induced by H_2_O_2_ and also for the capability of eliminating DPPH radicals. The results showed that all ethanolic extracts were efficient in antioxidant activities, reducing the action of H_2_O_2_ in the CP12 cell line. Compounds 44, 45, 46, 47, and 48 (Figure 5) demonstrated DPPH radical scavenging activity with IC_50_ values of 236.8 *μ*M, 156.3 *μ*M, 273.9 *μ*M, 281.1 *μ*M, and 60.9 *μ*M, respectively, with H_2_O_2_ concentrations ranging from 0.16 to 100 *μ*M. Lignan 48 showed the best antioxidant activity. Subsequent studies may apply these lignans for pharmacotherapeutic treatments of ALS and additional neurodegenerative diseases [105].

Sesamine (Figure 5), as reported previously, was also the object of the study of Shimoyoshi et al. They examined the possibility of sesamine affecting the cognitive decline in mice accelerated by senescence (SAMP10). Analytical methods showed changes in oxidative stress related to age in reactive brain carbonyl species (CRs) in mice treated with sesamine. The results showed that mice treated with sesamine obtained better results in tasks of avoidance and forced swimming with aging. Oxidative stress in the cerebral cortex and liver showed a reduction in mice treated with lignan. These results suggest that sesamine prevents brain dysfunction by antioxidative activity [106].

### 2.8. Quinones

Similar to the other secondary metabolites, quinones present in extracts have been reported in studies looking for ALS treatments and different neurodegenerative diseases [107–109].

**Figure 6:**
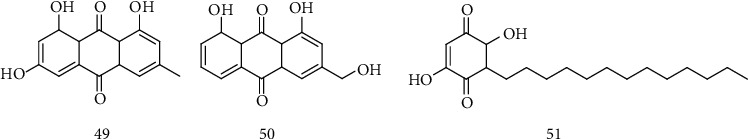
Structure of emodin (49), aloe-emodin (50) quinones found in the roots of the *Rheum officinale baill* plant, and rapanone (51), a benzoquinone isolated from the *Myrsine* plant.

One of these reports, presented by Lu et al., demonstrated the antioxidant effects of ethanolic extracts from the roots of the *Rheum officinale baill* plant of the *Polygonaceae* family, popularly known as rhubarb. Phytochemical analysis of the extracts demonstrated the presence of anthraquinones (emodine 49) and dianthraquinones (aloe-emodine 50) (Figure 6), in addition to other constituents. Rhubarb extract considerably decreased the number of neurons with condensed and fragmented DNA, especially at a concentration of 10 *μ*g/mL (at concentrations 2 and 5 *μ*g/mL the results were inferior). The study showed a protective role for rhubarb extract against cell oxidation, which may bring significant results in further studies in the treatment of ALS and other neurodegenerative diseases [109].

Vegás-Hernández and collaborators evaluated the antioxidant effects of rapanone (51) (Figure 6), a natural benzoquinone isolated from the *Myrsine* plant, against the chelation, iron removal activity, and protective potential against damage induced by *tert*-butyl hydroperoxide in mitochondria. The results showed the formation of complexes with irons II and III, oxidation of iron II, and peroxide radicals, inhibiting Fenton-Haber-Weiss reactions, effectively demonstrating the protective effect against oxidative stress at concentrations of 50 and 300 *μ*M. The results demonstrate the protective potential of rapanone against mitochondrial lipid peroxidation with IC_50_10.33 ± 1.29 at 50 *μ*M values better than the positive glycol-bis(2-aminoethylether)-*N*,*N*,*N*′,*N*′-tetraacetic acid (EGTA) IC_50_ 15.47 ± 2.01 *μ*M at 200 *μ*M. Thus, rapanona appears with pharmacological potential in the treatment of diseases that occur oxidative stress such as neurodegenerative diseases [110].

Studies with terpenoids, lignans, and quinones are more limited in the literature; however they exhibit greater depth of studies aimed at treating ALS. Unlike studies with tannins, these three metabolites, despite the few studies found, have advanced to *in vivo* studies, presenting significant results in different pathways. Few studies have reported the isolation of the main compound and do not report the extraction methodology transparently. Finally, despite having been carried out *in vivo*, the studies have not yet been reported or evaluated in clinical tests. It may be related to difficulties in isolation, since the extractive methods were not well understood in the study.

### 2.9. Saponins

The classic definition of saponins is based on its surface activity and its detergent properties, forming stable foams in water. Saponin is a class of glycosidic compounds that can be classified as steroidal saponins and triterpene saponins, which can form colloidal solutions that foam in water, like soap, if the mixture is stirred. Saponins can be found in the roots and leaves of plants and present antimicrobial, antibacterial, antiviral, anticancer, and antioxidants activities, in addition to its ability to stimulate the formation of collagen, a protein that plays a role in the healing process of wounds [111–113].

**Figure 7:**
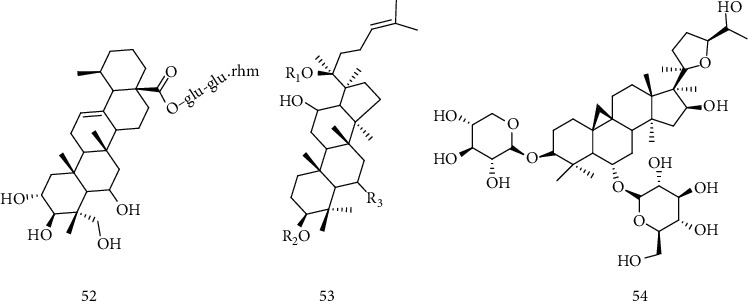
Chemical structure of madecassocide (52), ginsenosídeo (53), and astragalosídeo IV (54),

Many saponins have been reported for neuroprotection purposes, including madecassoside (52) [114–117], ginsenoside (53) [118–120], and astragaloside IV (54) [121] (Figure 7). Saponin's neuroprotection property is not correlated to a single process, but to a spectrum of independent and related processes, which promotes its ability to regenerate the neural network directly promoting cell survival, improving neurite growth, and recovering the activities of axons and synapses. These metabolites can also prevent neuron malfunctions by changing levels of neurotransmitters, receptors, and messengers [114, 121]. In this sense, madecassoside (52) is one of the bioactive compounds of the triterpenoid saponins isolated from *Centella asiatica* (L) *Urban*, reported with the potential to protect the degeneration of motor neurons and also increase the longevity of transgenic mice SOD1G93A with ALS [114].

In a model of ALS using a type of lipopolysaccharide-induced rats (LPS), two different doses of madecassoside were tested, 61.1 ± 11.0 and 185.6 ± 18.7 mg/kg/day, respectively. Compared to the control group (mice without madecassoside treatment), madecassoside failed to delay the onset of the disease, but can significantly prolong the mice's survival time by 11.4 and 9.4 days, respectively (*P* < 0.05) [113, 114]. Also, madecassoside inhibits the production of nitric oxide (NO) (a harmless component when present in small quantities, but undesirable when secreted in large quantities), prostaglandin E2 (PGE2), and additional inflammatory factors in RAW264.7 macrophage cells lipopolysaccharide-induced (LPS) and collagen-induced arthritis in a rat model, which could explain its neuroprotective and anti-inflammatory effect [113].

According to a study by Sasmita et al., madecassoside (52) is introduced as a potent antineuroinflammatory agent, which is able to influence genetic and protein components that are implicated in neuroinflammation and to reduce the intracellular levels of reactive oxygen species (ROS) by 56.84% in BV2 microglia cell line, in comparison to the group stimulated only with LPS. The results of the present study suggest that madecassoside successfully and significantly regulated the gene and protein expression of inducible nitric oxide synthase (iNOS), cyclooxygenase 2 (COX-2), signal transducer and transcription activator 1 (STAT1), and nuclear factor kappa B (NF-*κ*B), which are all proneuroinflammatory components. In addition, madecassoside significantly increased the gene expression of the anti-inflammatory component heme oxygenase 1 (HO^−1^) by 175.22% compared to the LPS group, suggesting that this compound is an important ally in the treatment of neurodegenerative diseases [115].

In another study by Liu et al., in which researchers evaluated the protective effect of madecassoside (52) on the cognitive process induced by LPS and neuroinflammation in rats, it was observed that the treatment with madecassoside (120 mg/kg, i.g.) for 14 days reduced LPS-induced neurotoxicity, decreasing cognitive deficiencies and suppressing the production of inflammatory cytokines, such as interleukin 1-beta (IL-1*β*), tumor necrosis factor alpha (TNF-*α*), and interleukin 6 (IL-6), through the activation of nuclear factor 2 signaling related to erythroid nuclear factor 2 (Nrf2). In addition, the treatment improved heme oxygenase (HO^−1^) protein levels, through the positive regulation of Nrf2 in LPS-stimulated neurotoxicity. Collectively, these results suggest that madecassoside is effective in preventing neurodegenerative diseases by improving memory functions due to its anti-inflammatory activities and activation of Keap1-Nrf2/HO^−1^ signaling [113].

Additional studies with madecassoside (52) extracted from *Centella asiatica* (CA) were also carried out evolving the evaluation of its potential to inhibit the enzyme Serina Racemase (SR), an enzyme responsible for converting L-serine into D-serine, an amino acid coagonist at glutamate N-methyl-D-aspartate (NMDA) receptors, whose hyperexcitation is involved in several neurodegenerative diseases, including ALS. The authors observed that the activity of the SR enzyme was significantly inhibited in the presence of CA extract. The CA extract at a concentration of 20 *μ*g/mL and 40 *μ*g/mL inhibited 85% and 99% of SR activity (*P* = 0.0001), respectively. The enzyme inhibition assay indicates that madecassoside is an inhibitor of human SR. The IC_50_ value for madecassoside is 26 *μ*M, which is the lowest IC_50_ among SR inhibitors reported up to date, giving this compound anti-inflammatory, neuroprotective, antiapoptotic, and antioxidative activity in animal models of ALS [116].

**Figure 8:**
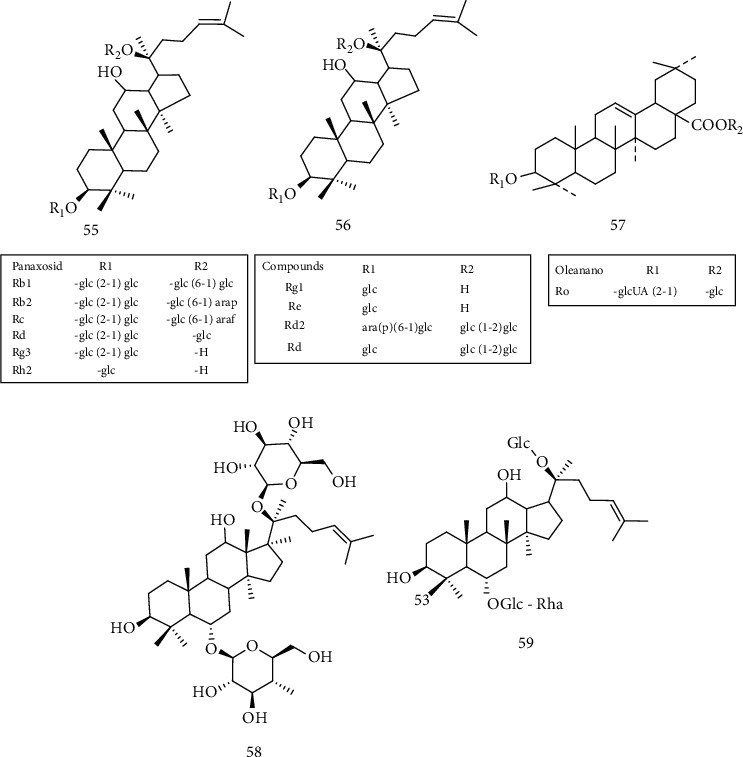
Chemical structure of (55), protopanaxatriol (56), Oleanano (57), ginsenoside Rg1 (58), and ginsenosídeo Re (59).

Ginsenosides are saponins found in the medicinal herb *Panax ginseng* that can be classified into three groups (Figure 8) based on structural differences: the protopanaxadiol group (55) (including Rb1, Rb2, Rb3, Rc, and Rd), the protopanaxatriol group (56) (as the Re, Rf, Rg1, and Rg2 derivatives), and the Oleanano group (as the Ro variant) (57) [117–119]. Different studies have shown that the components of *Panax L*., especially the ginsenosides Rb1, Rb2, Rb3, Rc, Rd, Re, Rg1, Rg2, and Rg3, have significant therapeutic effects in various neurological disorders such as memory, anxiety, depression, epilepsy, accident stroke, ALS, Alzheimer's disease (AD), Parkinson's disease (PD), and Huntington's disease [119]. Each ginsenoside molecule is potentially effective in the treatment of various diseases of the central nervous system (CNS). For example, the biologically active ginsenoside component Rg1 reduces reactive oxygen species (ROS) and cytotoxicity by negatively regulating proapoptotic proteins, neutralizing oxidative stress, resulting in neuroprotection of cells treated with lipopolysaccharides (LPS) [117, 118]. The ginsenosides present in ginseng have shown the potential to be an excellent compound for the treatment of neurodegenerative diseases, possibly its neuroprotective effect occurs due to the modulation of neuronal calcium channels. Some studies suggest that ginsenoside Rg1 (58) (Figure 8), panaxatriol saponin, reduces the production of reactive oxygen species (ROS), releases cytochrome c in the cytosol, inhibits caspase3 activity, and lowers nitric oxide production (NO), reducing the level of inducible protein of nitric oxide synthase (iNOS), in addition to being reported rescue of cellular damage caused by hydrogen peroxide through the negative regulation of the NF-*κ*B signaling pathway, as well as activation of the AKT and ERK1/2 [118, 122, 123].

A preliminary study by Jiang et al. [58] with ginseng root showed beneficial effects in an ALS model using SOD1G93A transgenic mice, portraying a delay in disease onset and a prolonged survival rate. This study, combined with the anti-inflammatory, antioxidant, antiapoptotic, and immunological effects on the CNS reported in the literature, encouraged Ratan et al. [119] to evaluate the effects of the ginsenoside Re (G-Re) (59) (Figure 8) on neuroinflammation and its action on human superoxide dismutase 1 (SOD1) through the administration of 2.5 *μ*g/g of the compound in symptomatic ALS hSOD1G93A mice. The authors observed that treatment with G-Re reduced the loss of motor neurons and the expression of Iba-1 (ionized calcium binding adapter molecule 1), a microglia-specific calcium-binding protein, in the spinal cord of transgenic mice hSOD1G93A, indicating a possible neuroprotective effect. Besides, compared to hSOD1G93A mice of the same age, those treated with G-Re showed a significant reduction in the expression of proinflammatory proteins, like the CD14 protein and TNF-*α* related to the TLR4 signaling pathway. G-Re administration also led to a decrease in phospho-p38 protein levels related to cell death and had an antioxidant effect by reducing the expression of HO^−1^. These results suggest that ginsenoside-Re has a potent antineuroinflammatory effect on ALS, inhibiting the TLR4 pathway.

Astragaloside IV (AST-IV) (54) is a small saponin and the main active component in *Astragalus membranaceus*, holding anti-inflammatory, antiviral, antiaging, immunomodulatory, and neuroprotective effects. The neuroprotective effect of this compound was observed in neural microglia cells, in which the presence of AST-IV in the concentration of 1 to 5 *μ*mol/L protected the microglia from death caused by LPS and negatively regulated the release of proinflammatory mediators, including interleukin IL-1*β*, IL-6, tumor necrosis factor *α* (TNF-*α*), and nitric oxide, as well as the expression of Toll-like 4 receptors (TLR4), MyD88, and nuclear factor *κ*B (NF-*κ*B) of these cells. These results indicate that AST-IV exerts an anti-inflammatory effect on microglia, possibly by inhibiting the TLR4/NF-*κ*B signaling pathways and protects neurons from microglia-mediated cell death by converting the inflammatory M1 microglia into a M2 anti-inflammatory phenotype, indicating the potential of this compound for the treatment of neurodegenerative diseases [120].

The evidence of possible beneficial effects during *in vitro* and *in vivo* studies, shown in the studies above, is a starting point for the achievement of new therapeutic alternatives in the treatment of ALS. However, it is important to note that such studies demonstrate a lack of methodological standardization of the samples used, ranging from commercially obtained saponins [113, 117, 120, 124] to plant extracts [116]. The lack of standardization of the samples can influence the reproducibility of the results obtained in these initial tests.

### 2.10. Methylxanthines

**Figure 9:**
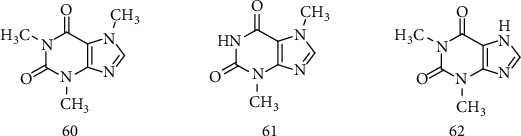
Chemical structure of methyl xanthine: cafeíne (60), theophylline (61), and theobromine (62).

Methylated xanthines (methylxanthines) are heterocyclic organic compounds that are methylated derivatives of xanthine, therefore comprising coupled rings of pyrimidinedione and imidazole. They are present in almost 100 species of 13 orders from the plant kingdom, including tea (*Camellia sinensis L*.), coffee (*Coffea* sp.), and cocoa (*Theobroma cacao L*.), being normally included in the daily human diet in many drinks and foods extremely common, such as coffee, tea, cocoa, yerba mate, and cola. Caffeine (60), theophylline (61), and theobromine (62) (Figure 9) are the main methylxanthines available from natural sources [125, 126].

Methylxanthines have been widely used as therapeutic agents, in a range of medicinal fields, such as CNS stimulants, bronchodilators, coronary dilators, diuretics, anticancer adjuvant treatments, and, more recently, they have been suggested with a potential beneficial health effect in the treatment of neurodegenerative diseases, cardioprotection, diabetes, and fertility [126].

Caffeine is a methylxanthine that nonselectively antagonizes adenosine receptors and is the most widely used psychoactive substance in the world. Its chronic consumption has proven to be protective against neurodegenerative diseases, such as Parkinson [127], Alzheimer [128], and ALS [129]. For many years, it was believed that caffeine consumption could be associated with an increased risk of neurodegenerative diseases. However, Fondell and colleagues [129] performed a longitudinal analysis based on more than 1,010,000 men and women in five large cohort studies, in which they evaluated the association between caffeine, coffee, and tea consumption and ALS risk. The results showed that a total of 1,279 cases of ALS were documented during an average of 18 years of follow-up. Caffeine intake was not associated with the risk of ASL; the combined multivariate adjusted risk (RR) ratio was 0.96 (95% CI 0.81-1.16). Similarly, neither coffee nor tea has been associated with ASL risk. Therefore, the results of this large study did not demonstrate any associations of caffeine or caffeine drinks with the risk of ASL [130, 131].

The data found by Fondell and collaborators [129] reaffirm the results obtained by Herden and Weissert [132] when investigating in 377 patients with newly diagnosed ALS the relationship between coffee consumption and ALS risk in which they evidenced an inverse correlation between the consumption of coffee and the risk of disease.

Onatibia-Astibia et al. and Sonsalla et al. [125, 127] report that caffeine consumption improves cognitive function, prevents neurodegeneration, and restores neuronal plasticity and mitochondrial production in middle-aged rats and in the APPswe mouse model of Alzheimer's disease. Regarding ALS, caffeine is believed to be neuroprotective, antagonizing A_2A_ adenosine receptors in the brain and thus protecting motor neurons from excitotoxicity [128, 129]. In this sense, Monteiro et al. [126], Kolahdouzan and Hamadeh [130], and Herden and Weissert [132] also reinforce the beneficial effects of caffeine consumption in improving motor activity in the context of neurodegenerative diseases through mechanisms of neuroprotection and neurorestoration by trophic proteins, suggesting that caffeine is a promising compound for the treatment of neurodegenerative diseases.

Caffeine is the most studied methylxanthine concerning its effect on neurodegenerative diseases. According to the studies with patients reported above, the consumption of caffeine has shown a protective effect in patients with ALS. On the other hand, there is a lack of evidence on the neuroprotective mechanism of action of this compound, and it is necessary to expand research in this area, using *in silico*, *in vitro*, and *in vivo* experiments, not only to elucidate the effect of caffeine on neurodegenerative diseases, but also to investigate new methylxanthines that may show a potential effect on these diseases. So these structures can be explored for the design of new compounds that may be more promising, facilitating the understanding of their possible mechanisms of action.

### 2.11. Glucosinolates

**Figure 10:**
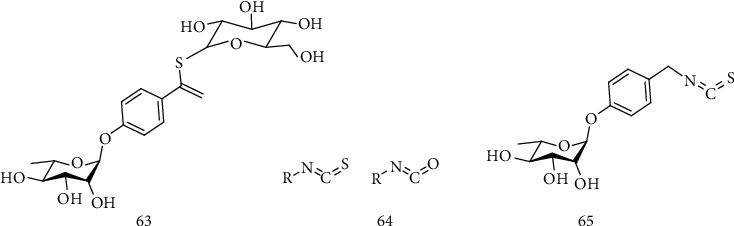
Chemical structure of 4-O-(*α*-*L*-ramnopyranosyloxy)-benzyl (GMG) (63), isothiocyanates (ITCs) (64), and 4-(*α*-*L*-rhamnosyloxy)-benzylisothiocyanate) (GMC-ITC) (65).

Glucosinolates are abundant compounds in the *Moringa* species, mainly in the *Moringa oleifera*, having been reported to have anti-inflammatory, antioxidant, anticancer, and antidiabetic activities. The most abundant glucosinolate present in the species is 4-O-(*α*-*L*-ramnopyranosyloxy)-benzyl glucosinolate, also known as glucomoringin (GMG) (63). Glucosinolates and related hydrolytic products, isothiocyanates (ITCs) (64), have attracted researchers due to their neuroprotective effect [133–136]. ITCs are phytochemicals containing sulfur and nitrogen obtained from glucosinolates after myrosinase action. Myrosinasin-catalyzed hydrolysis at neutral pH of GMG releases the biologically active compound 4-(*α*-*L*-ramnosiloxy)-benzylisothiocyanate (GMG-ITC) (65) (Figure 10) [135, 136].

Many studies have revealed the benefits of consuming vegetables from *Brassicaceae* and *moringaceae*, which are the main sources of ITCs. These compounds are considered to contribute to the massive reduction of risk for the development of neurodegenerative diseases, due to their antiamyloidogenic, antioxidant, and anti-inflammatory properties. The preventive and treatment capacity of ITCs for NDDs is being extensively explored over the last years [135].

In this sense, Jaafaru and collaborators [137] evaluated the neuroprotective activity of GMG-ITC (65) by eliminating ROS in SH-SY5Y neuroblastoma cells (ATCC® CRL-2266 ™), observing that the presence of GMG-ITC (1.25 *μ*g/mL) before the development of the oxidative stress condition decreased the expression of cyt-c (cytochrome C), p53 (also called TP53 gene and tumor protein p53 gene), Apaf-1 (apoptosis-activating factor 1), Bax (BCL-2-associated protein X), CASP3, CASP8, and CASP9 (proteins involved in the caspase pathway) with simultaneous positive regulation of the Bcl-2 gene (B cell lymphoma protein 2) in the mitochondrial apoptotic signaling pathway. In view of these results, the authors suggest that pretreatment with GMG-ITC can relieve the condition of oxidative stress in neuronal cells, reducing the level of ROS production and protecting cells against apoptosis through possible neurodegenerative pathways of disease [137].

In another study, Galuppo et al. [136] evaluated the therapeutic efficacy of GMG-ITC against ALS *in vivo* using SOD1tg mice, which physiologically develop SOD1G93A at around 16 weeks of age and can be considered a genetic model of the disease. The rats were treated once a day with GMG (10 mg/kg) bioactivated with myrosinase (20 *μ*L/rat) by intraperitoneal injection for two weeks before the onset of the disease, and the treatment was prolonged for another two weeks before sacrifice. The results showed statistically significant differences between mice treated and untreated with GMG-IT (*P* = 0.003), demonstrating that the administration of GMG-IT was able to delay the onset of the disease for approximately two weeks, probably due to its immunomodulatory, anti-inflammatory, antioxidant, and antiapoptotic effects, suggesting that this compound could be a potential drug for the treatment of this disease.

Glucosinolates have been extensively studied in recent years due to their anti-inflammatory and antioxidant potential. However, few studies in the literature report their action on neurodegenerative diseases, such as ALS. Preliminary studies in both cells and animals show neuroprotective effects of these compounds, but the mechanism of action and the extent of the effects have yet to be further investigated in more robust preclinical and clinical studies.

### 2.12. Fatty Acids

The growing amount of researches involving neurodegenerative diseases shows that neurons are particularly vulnerable to oxidative damage due to their high content of polyunsaturated fatty acids in the membranes, high oxygen consumption, and weak antioxidant defense [138]. Fatty acids are essential lipid molecules to support the structural integrity of cell membranes, providing energy and assisting in signaling pathways. Studies show that a higher intake of polyunsaturated fatty acids *ω*3, considered neuroprotectives, was associated with a reduced risk of ASL [139].

**Figure 11:**
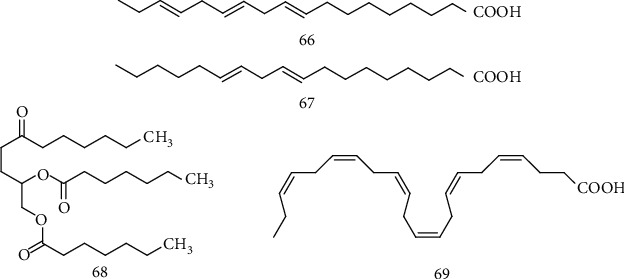
Chemical structure of omega-3 (66), omega-6 (67), triheptanoin (68), and docosahexaenoic acid (DHA) (69).

On the other hand, Boumil et al. estimated the impact of omega-3 (*Ω*-3) (66) and omega-6 (*Ω*-6) (67) (Figure 11) in the function of the motor neuron in mice expressing the mutant human superoxide dismutase-1 (SOD1). The authors noted that supplementation with *Ω*-3 and *Ω*-6 equivalents hastened the pathology and death of motor neurons; however, 10x *Ω*-6 with no change in *Ω*-3 (1 mg/kg) significantly delayed pathology in motor neurons, including preservation of minor motor neuron function during the terminal stage, suggesting that a critical balance of *Ω*-6 and *Ω*-3 may temporarily preserve motor neuron function during the terminal stages of ALS, which could provide a substantial improvement in the quality of life of the affected individuals [140].

Assessing the therapeutic potential of fatty acids for the treatment of ALS, Tefera and collaborators [141] observed the effects of triheptanoin fatty acid (68) (Figure 11), a heptanoate triglyceride, in the treatment of mice that overexpress the SOD1G93A human gene mutant. They observed that rats fed the SF11-028 diet (composed of ingredients typical of other rat diets), modified with 35% oral triheptanoin, starting at P35 (35th day), showed attenuation in the loss of motor neurons at 70-day old by 33%. This result, combined with the pressure strength of the hind limbs, which demonstrated a delay in the loss of strength in hSOD1G93A mice treated with triheptanoin, suggests that this compound delays the loss of motor neurons and the appearance of motor symptoms in mice.

According to Días-Amarilla et al., nitro-fatty acids (NO_2_-FA) are electrophilic signaling mediators formed in tissues during inflammation, capable of inducing cytoprotective pathways and pleiotropic antioxidants, including the regulation of genes responsive to factor 2 (Nrf2) related to the erythroid nuclear factor. These researchers demonstrated that nitro-arachidonic acid (NO_2_-AA) or nitro-oleic acid (NO_2_-OA) administered in astrocytes that express hSOD1G93A bound to ALS at a concentration of 5 *μ*M induces an antioxidant effect through the activation of Nrf2 concomitantly with the increased levels of glutathione. In addition, the treatment of astrocytes expressing hSOD1G93A with NO_2_-FA prevented its toxicity to motor neurons, proving its antioxidant effect on astrocytes capable of preventing the death of motor neurons in an ALS culture model [142].

Trostchansky and collaborators also evaluated the response of transgenic SOD1G93A mice with ALS after the treatment with 16 mg/kg/day of nitro-oleic electrophilic acid (NO_2_-OA) subcutaneously. It was observed that NO_2_-OA significantly improved the grip strength and performance on rotarod (also called rotating bar that assesses motor coordination and balance) compared to animals treated with vehicle or oleic acid (AO), suggesting NO_2_-OA as a promising therapeutic compound [143].

Another fatty acid of importance in ALS is docosahexaenoic acid (DHA) (69) (Figure 11), an essential fatty acid that modulates the main functions of the nervous system, including neuroinflammation, and regulation of pre- and postsynaptic membrane formation, being reduced in patients with amyotrophic lateral sclerosis and preclinical murine models [144]. Based on this information, Torres et al. [144] performed dietary supplementation of DHA in a murine model fASL B6SJL-Tg (SOD1 ∗ G93A), in which they observed that a diet rich in DHA significantly increases the survival of male rats by 7% (average of 10 days over 130 days of life expectancy) and delays motor dysfunction (based on stride length) and loss of weight associated with ALS transgene (*P* < 0.01). Also, DHA supplementation led to an increase in the profile of anti-inflammatory fatty acids (*P* < 0.01), a lower concentration of circulating proinflammatory such as the cytokine TNF-*α* (*P* < 0.001 in males), reduced immunoreactivity to markers of oxidative DNA damage (8-oxodG) in the lumbar spinal cord (LSC), and preserved the number of motor neurons compared to untreated. The literature also suggests the antioxidant action of fatty acids as the main mechanism of action.

Due to the importance of fatty acids for the structure and integrity of cell membranes, these compounds have been investigated with a potential neuroprotective effect. Studies in murine models have shown a beneficial effect obtained by the supplementation with fatty acids, such as DHA, omega-3 (*Ω*-3), and omega-6 (*Ω*-6). Nonetheless, it is important to emphasize that the amount to be supplemented is not yet well established. Some studies have suggested that the neuroprotective effect is derived from a balance between fatty acids [140]. Therefore, further research is required to establish the neuroprotective potential, the neuroprotective mechanism, and the amount of fatty acids to be used to ensure this effect.

## 3. Conclusions

Unfortunately, there is still no effective and/or curative treatment for the disease, and the few drugs on the market are intended to only slow the progress of the disease. Among the most promising treatment alternatives, the effectiveness of antioxidants can be highlighted, since the disease is directly related to oxidative stress and cell death. In this context, the relevance of natural products with potential antioxidant action was reported in this review, emphasizing on oxygenated and nitrogenous compounds that are capable of acting in redox balance, attenuating or decreasing the impact of these effects on neuronal and motor cells. Among them, we highlight polyphenols, flavonoids, coumarins, and alkaloids as main metabolites, as well as unsaturated hydrocarbons, such as fatty acids and their esters, which are part of the diet worldwide and are considered promising alternatives. The mechanisms of action associated with the activity of these metabolites are not yet entirely elucidated, but researches point out that natural products capable of regulating redox effects are fundamental for cellular processes, maintaining an appropriate environment for metabolic activities and healthy functioning, as they present, in some cases, low collateral effects and multiple targets. Nevertheless, there are still few studies related to toxic effects, mechanisms of action, and strategies of molecular modifications using these prototypes through total synthesis or semisynthesis, which could lead to further preclinical and clinical trials, and eventual obtainment of safe and effective compounds that can improve the ALS patient's outcome.
